# Correction: Cui et al. Anticancer Peptides Derived from Aldolase A and Induced Tumor-Suppressing Cells Inhibit Pancreatic Ductal Adenocarcinoma Cells. *Pharmaceutics* 2023, *15*, 2447

**DOI:** 10.3390/pharmaceutics18070808

**Published:** 2026-06-30

**Authors:** Changpeng Cui, Qingji Huo, Xue Xiong, Kexin Li, Melissa L. Fishel, Baiyan Li, Hiroki Yokota

**Affiliations:** 1 Department of Pharmacology, School of Pharmacy, Harbin Medical University, Harbin 150081, China; cuich@iu.edu (C.C.); qinghuo@iu.edu (Q.H.); xiongxue@iu.edu (X.X.); kexinli0104@gmail.com (K.L.); 2 Department of Biomedical Engineering, Indiana University Purdue University Indianapolis, Indianapolis, IN 46202, USA; 3 Department of Pediatrics, Wells Center for Pediatric Research, Indiana University School of Medicine, Indianapolis, IN 46202, USA; mfishel@iu.edu; 4 Department of Pharmacology and Toxicology, Indiana University School of Medicine, Indianapolis, IN 46202, USA; 5 Indiana University Simon Comprehensive Cancer Center, Indianapolis, IN 46202, USA; 6 Department of Pediatrics, Indiana Center for Musculoskeletal Health, Indiana University School of Medicine, Indianapolis, IN 46202, USA

## Error in Figure

In the original article [1], there was a mistake in Figure 3B as published. During figure preparation, an incorrect image was inadvertently used in the PANC198 panel. The corrected Figure 3B appears below. The authors apologize for any inconvenience caused and state that the results and scientific conclusions are unaffected. This correction was approved by the Academic Editor. The original publication has also been updated.

## Figures and Tables

**Figure 3:**
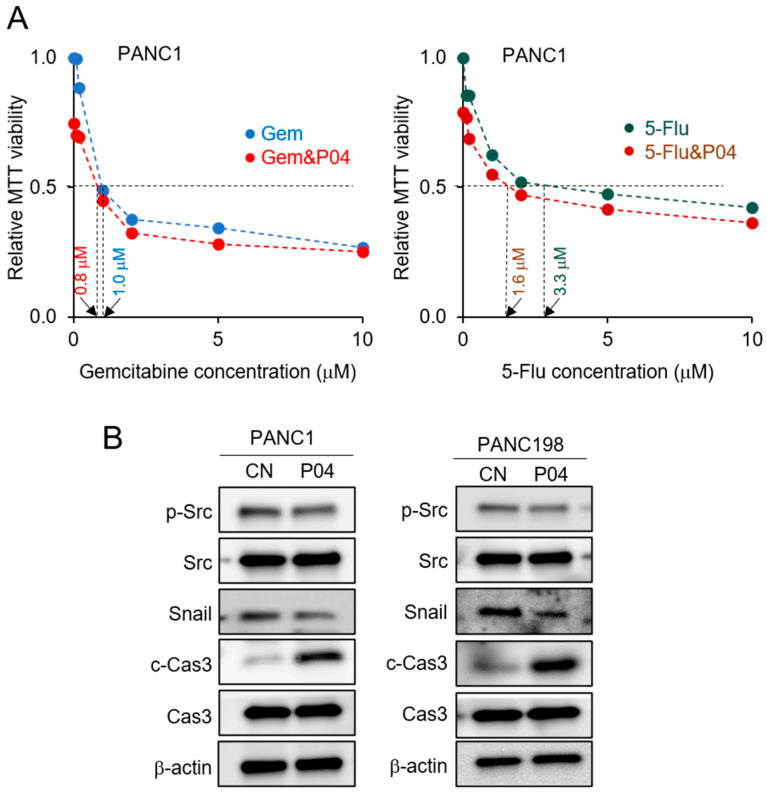
Inhibitory effects of P04 in combination with gemcitabine and 5-Flu. CN = control, Gem = gemcitabine, and 5-Flu = 5-fluorouracil. (**A**) Additive anti-tumor effects of P04, together with gemcitabine or 5-Flu; (**B**) decrease in the levels of p-Src and Snail, as well as an increase in cleaved caspase 3 (c-Cas-3) in PANC1 and PAN198 PDAC cells in response to 25 μg/mL of P04.
