# Supplementary material for: Anticancer Peptides Derived from Aldolase A and Induced Tumor-Suppressing Cells Inhibit Pancreatic Ductal Adenocarcinoma Cells
Source: Pharmaceutics. 2023 Oct 11;15(10):2447. doi: 10.3390/pharmaceutics15102447 (PMC10610494; doi:10.3390/pharmaceutics15102447)
Supplement: Supplementary file 1 [file pharmaceutics-15-02447-s001.zip › pharmaceutics-2612479-supplementary.pdf]

## Supplementary Information

## Supplementary Tables

Supplementary Table S1. Anti-cancer peptide candidates.

| Peptide | Sequence |
|---|---|
| P01 | SerGluThrAlaProAlaAlaProAlaAlaProAlaProAlaGluLys |
| P02 | AlaGluGluTyrGluPheLeuThrProMetGluGluAlaProLys |
| P03 | HisValPheGlyGluSerAspGluLeuIleGlyGlnLys |
| P04 | IleGlyGluHisThrProSerAlaLeuAlaIleMetGluAsnAlaAsnValLeuAlaArg |
| P05 | AlaAspAspGlyArgProPheProGlnValIleLys |
| P06 | GlyAlaGlyThrGlyGlyLeuGlyLeuAlaValGluGlyProSerGluAlaLys |
| P07 | ValGluProGlyLeuGlyAlaAspAsnSerValValArg |
| P08 | AsnSerAsnLeuValGlyAlaAlaHisGluGluLeuGlnGlnSerArg |
| P09 | AlaAlaGlyThrLeuTyrThrTyrProGluAsnTrpArg |
| P10 | PheAlaAlaAlaThrGlyAlaThrProIleAlaGlyArg |

Supplementary Table S2. MTT-based viability of 4 pancreatic cancer cell lines in response to 10 anticancer peptide candidates at 25 mg/mL.

| Cell line | P01 | P02 | P03 | P04 | P05 | P06 | P07 | P08 | P09 | P10 |
|---|---|---|---|---|---|---|---|---|---|---|
| PAN198 | 0.92 | 0.94 | 0.96 | 0.79 | 0.87 | 0.98 | 0.95 | 1.03 | 1.01 | 0.98 |
| PANC1 | 0.97 | 0.95 | 0.97 | 0.76 | 0.86 | 0.94 | 0.92 | 0.95 | 0.89 | 0.91 |
| PA03C | 0.97 | 0.95 | 0.91 | 0.85 | 0.85 | 1.00 | 0.94 | 0.95 | 0.94 | 0.97 |
| ASPC1 | 0.93 | 0.89 | 0.98 | 0.81 | 0.87 | 0.97 | 0.96 | 1.04 | 1.02 | 0.99 |
| Mean | 0.95 | 0.93 | 0.95 | 0.80 | 0.86 | 0.97 | 0.94 | 0.99 | 0.97 | 0.96 |

Supplementary Table S3. Hydrogen-bonding interaction in EGFR-P04 complex.

| EGFR Receptor Residue | P04 Ligand Residue | Interaction Consistituents | Distance (Å) | Type |
|---|---|---|---|---|
| B:ASN210 | P04:LEU171 | B:ASN210:N - P05:LEU171:O | 3.0174 | Conventional |
| B:CYS207 | P04:ARG173 | P04:ARG173:NH1 - B:CYS207:SG | 2.8805 | Conventional |
| A:SER196 | P04:GLU166 | A:SER196:CB - P05:GLU166:O | 2.6857 | Carbon |
| B:THR249 | P04:GLY155 | P04:GLY155:CA - B:THR249:OG1 | 3.0735 | Carbon |

**Supplementary Figure**

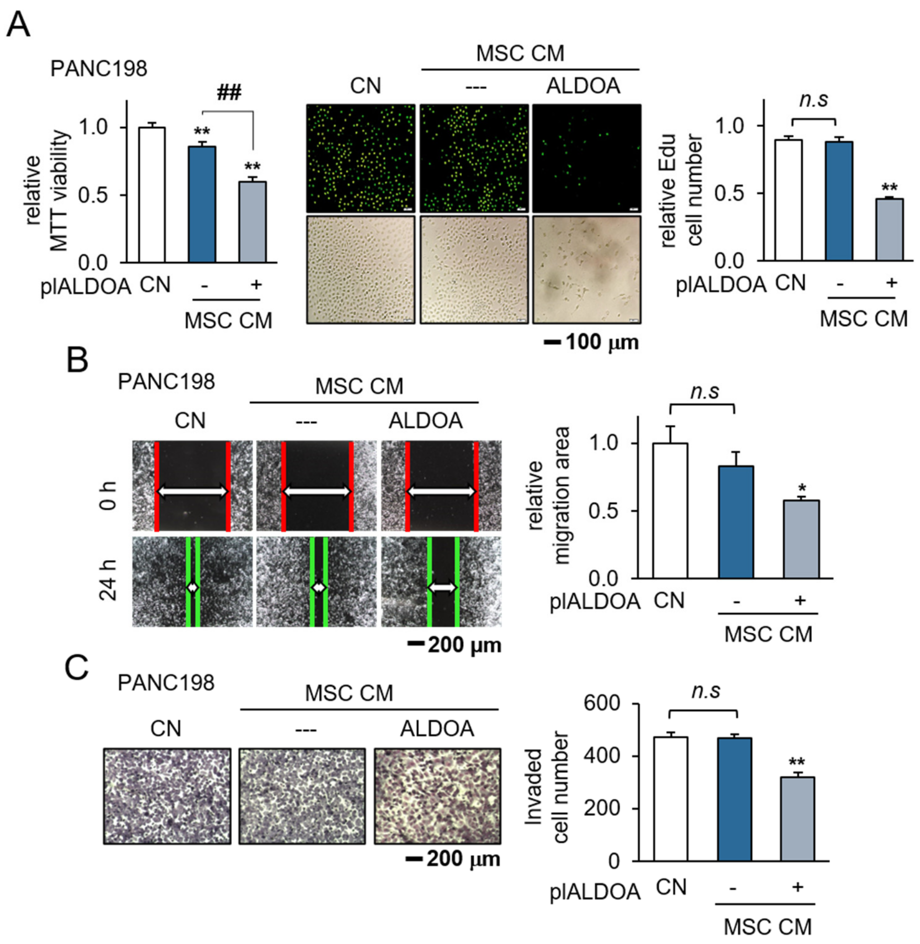


**Supplementary Figure S1.** Generation of iTSCs by overexpressing ALDOA in MSCs. CN = control, pl = plasmid transfection, MSC = mesenchymal stem cell, and CM = conditioned medium. The single and double asterisks indicate $p < 0.05$ and 0.01, respectively. (A-C) Reduction in MTT-based viability, EdU-based proliferation, scratch-based motility, and transwell invasion, respectively, in PANC198 cells, by ALDOA-overexpressing MSC-derived conditioned medium.

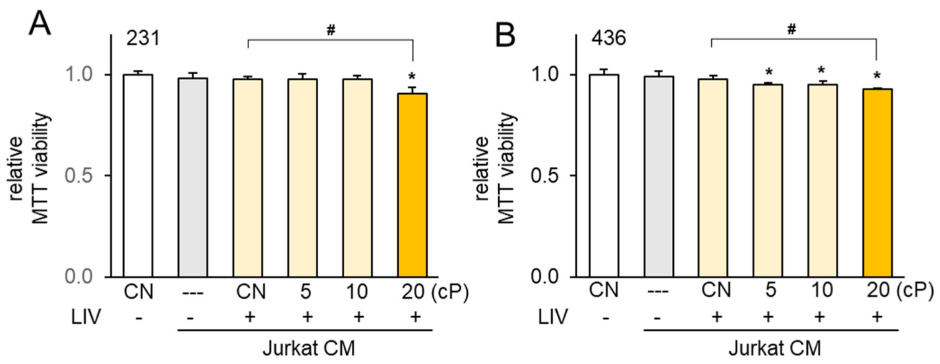


**Supplementary Figure S2.** Generation of iTSCs from LIV-treated Jurkat cells at different viscosity. CN = control, CM = conditioned medium, and cp = centipoise (viscosity unit). The single asterisk and hashtag indicate $p < 0.05$ to CN and LIV CN, respectively. (A&B) Reduction in MTT-based viability in MDA-MB-231 and MDA-MB-436 cells by LIV-treated Jurkat-derived CM.
